# Management of expatriate medical assistance in Mozambique

**DOI:** 10.1186/1478-4491-4-26

**Published:** 2006-12-02

**Authors:** Ferruccio Vio

**Affiliations:** 1 HIV/AIDS Care & Treatment Team, Health Alliance International, Rua Emília Dausse 17, Maputo, Mozambique

## Abstract

This paper discusses how Mozambique coped with the health system needs in terms of specialized doctors since independence, in a troubled context of war, lack of financial resources and modifying settings of foreign aid. The Ministry of Health (MOH) managed to make up for its severe scarcity of specialist MDs especially through contracting expatriate technical assistance. Different scenarios, partnerships and contract schemes that have evolved since independence are briefly described, as well as self-reliance option possibility and implications. Lessons learned about donor initiatives aimed at contracting specialists from other developing countries are singled out. The issue of obtaining expertise and knowledge in the global market as cheap as possible is stressed, and realistic figures of cost planning are highlighted, as determined by the overall health system necessities and budget limitations.

## Background

Shortly after independence (1975), the mass repatriation of the Portuguese cadres working in the Health System left the MOH with the urgent need of finding specialized medical doctors (MD) for its referral hospitals. In 1972 there were 289 MDs in the country [1]. In 1976, only about 60 MDs had remained [2]. The solution was found thanks to the assistance of the socialist countries, which promptly provided a contingent of mainly Russian and Cuban doctors. The political and economic collapse of the socialist block after 1990 caused a new crisis, threatening to leave hospitals without specialized MDs. After a rather chaotic period, the situation improved in 1996, through the activation of a pool funded by Switzerland, the Netherlands and Norway, which granted salaries for senior specialists, mainly from the former USSR republics, and kept the best of them in place. Under what was called the "pooling agreement", the MOH identified the need for personnel and took care of selection, supervision and assessment, while UNDP administered the contracting and payment of salaries. Meanwhile, national specialists were slowly increasing in number, but they continued to be insufficient to satisfy the public system needs. Moreover, virtually all of them were working in Maputo. As a way to encourage the few national medical specialists to work outside the capital, the MOH and Switzerland agreed a salary scheme (known as 'topping up'), which filled the gap between the public sector and expatriate salary scales working out of Maputo. In the meantime, bilateral cooperation still held a key role; in 1999, of a total of 406 MDs holding clinical posts, there were 204 foreigners abiding by different kinds of contract [3], most of them through inter-governmental agreements with Cuba and, to a lesser extent, China and Nigeria. This state of affairs remained almost unchanged until 2004.

The health sector in Mozambique is heavily dependent on external financial support (about 60% of the budget in 2004), while donors are progressively dropping financial mechanisms aimed at particular health programs or sectors for a broader policy of budget support. Dependence could increase with the expected worsening of the AIDS epidemic. The new AIDS program in Mozambique, aimed at rapidly expanding the access to ARV treatment for AIDS-patients, will create more demand for MDs, in a country where the estimated number of HIV-positive people presently amounts to 1 400 000. The total salary bill is increasing because of the growth of the entire health workforce (to cope with the new demand brought by AIDS) and because of a faster increase of graduated and middle level cadres [4].

### Specialised MDs in the national health system (NHS)

More than 90% of the health system is made up of a multitude of minuscule health centres and posts with very basic resources, and managed by elementary and basic level health personnel. In spite of figures of MD per capita in Mozambique being among the lowest in the world, work load for MDs and specialists working in the country's few sizeable hospitals does not look particularly high, at least not higher than what may be found in other sub-Saharan African Countries. [5,6].

Table 1 shows the strong concentration of specialist MDs in Maputo, most of them working at the capital Central Hospital (which employs 143). Many of the national ones work part-time in the private sector. The Maputo Central Hospital, by far the biggest health facility and the main centre for the clinical training of students from the State Medical School or in post-graduation training courses, is able to autonomously manage substantial funds (currently about USD 8 000 000 per year) obtained through the provision of semi-private services. This situation is unique in the NHS. Most of the money is used to pay incentives to the hospital workers and to hire new ones; in both instances, MDs are the main beneficiaries[7]. Moreover, the majority of the country's few private for-profit medical services are located in Maputo.

**Table 1:** Distribution of specialists, per region, Mozambique, 2004

		**Percentage**	
		
**Provinces**	**Specialists**	**Population**	**Hospital beds**
Town of Maputo	173	7.6	15.1
Other southern provinces	20	18.6	23.7
Central provinces	54	20.8	34.9
Northern provinces	51	53.6	26.3
*Total*	298	100	100

Table 2 shows that most of the posts outside the capital are occupied by foreign specialists. Nearly all 23 nationals who work in the same places as well benefit from the topping up scheme. It was shown that the scarcity of specialists is worsened still by the uneven distribution between Provincial Hospitals of the same level, especially when compared to the work load of each hospital, as illustrated in Table 3[8].

**Table 2:** MDs within and outside the capital, Mozambique, 2004

	**Foreigners**		**Nationals**		
		
**Provinces**	**Specialists**	**GP**	**Specialists**	**GP**	**Total**
Maputo	96	5	77	136	314
Outside Maputo	102	19	23	181	325
*Total*	198	24	100	317	639

**Table 3:** Work load per MD in 2003 in Mozambique's ten largest hospitals

**Hospital**	**Beds**	**Beds per MD**	**Occupied bed days per MD**	**Deliveries per gynaecologist**
Lichinga	186	17	5809	3712
Pemba	224	19	4472	1793
Nampula	313	10	4180	4049
Quelimane	457	22	5986	936
Tete	324	23	5976	3241
Chimoio	371	25	7850	2248
Beira	746	15	4799	1723
Inhambane	230	23	5350	2134
Xai-Xai	222	28	9553	3004
Maputo	1499	6	1740	829
*Total/average*	4572	11	3307	1138

### Pooling contract mechanism

Most specialists hired through the pooling mechanism are senior cadres with a long in-country experience, working out of the capital, which puts their cost relatively high, but rewards the health system with higher reliability. The number of doctors paid by this scheme oscillated between 50 and 60 annually, from 1996 to March 2000, the year in which donors withdrew their support from MD pooling [9]. In 2000 a study found that the pooling for specialized MDs and topping up greatly improved the overall situation, especially in previously neglected hospitals in the Northern and Central regions. The programmes 'enabled the NHS to select competent/productive cadres through transparent and competitive processes ... ensuring loyalty and discipline' [3]. It was also recognized that the new contractual environment improved productivity and human relationships of the MDs coming from the ex-socialist countries. Afterwards, the responsibility for contracting foreign MDs fell directly on the MOH managers and the bill was assumed by the State Budget, which is, in turn, is heavily financed by donors through the general budget support mechanism. However, spending difficulties due to weakness in financial management and to conflicting interests between the MOH directorates made the contracting of foreign specialists a rather troubled matter in the last years.

Pooling was unanimously considered a simple and successful initiative. Its dismissal was blamed, among others, 'on strained communication between the MOH and financers, lack of consensus between donors, poor coordination inside the MOH, unclear roles of involved parties and unrealistic expectations about the quick replacement of foreign physicians with national ones' [9].

### Topping up

The MOH and the Swiss Development Cooperation (SDC) have agreed a 'topping up' for national specialized MDs since 1997. It allows the MOH to have senior national cadres in all referral hospitals, with tasks of enforcing therapeutic and procedural national norms, coordination, supervision and teaching. Between 11 and 15 national specialists were contracted annually under this scheme until 2002. Currently their number is increasing. By topping up, national specialists could gradually replace expatriate ones contracted by the previous pooling agreement. A Sector Wide Approach (SWAp) financial scheme replaced the SDC as topping up financer in 2004; more posts are expected to be filled (25) by the gradually increasing number of newly trained specialists.

### Government bilateral agreement (GBA)

Direct Government agreements offer the advantage of lower transactional costs compared to individual contracts. On the side of Mozambique the only duty is to identify the needed specialities and to map the vacancies nationwide. However, it implies a very centralized process, where the headquarters makes a national plan and contracts the MD on behalf of every hospital involved. This approach seems at odds with the public sector reforms undertaken in Mozambique, directed to allow more autonomy in fund and resource management at lower levels of decision making. As elsewhere in health management, it is difficult to define a fair trade-off between efficiency gains and a sound management built on local capacity. Moreover, as in other African countries [10], almost all MDs contracted through government agreements (74 people) are Cubans. This option is based on the traditional friendship between the two countries and offers the advantage of a fast adaptation of the MDs, because of similarities of language and climate. However, this situation also creates some degree of dependency on a single source of supply without the possibility of exercising much control. In order to counter-balance some of the possible shortcomings, the government has chosen to diversify the recruitment countries and thus assure itself of a maximum of control over the supply situation.

### Individual contracts

Most doctors under this scheme come from developing countries, had been previously contracted under a GBA, and are continuing on an individual basis after their first contract terms came to an end. A very small fraction is made up of Portuguese doctors who remained in Mozambique after independence, or by MD catholic priests willing to work within the NHS.

In sum, a kind of osmosis exists between the different categories of expatriate specialists. Part of those contracted through bilateral agreements, after fulfilling their terms, manage to remain in Mozambique, shifting to 'ex-pooling' mechanisms or other kinds of individual contracts and/or, in some cases, to private practice. Thus, through a process of selection between expatriate specialists and a group of long-term residents, culturally and socially integrated MDs emerged as a structural and considerable component of the medical workforce in Mozambique.

Table 4 compares the financial burden of each kind of contract. Administrative costs, such as those associated with contract writing are higher for individual contracts. However, housing and travel costs are greater for the MDs hired through bilateral agreement than for individually contracted MDs.

**Table 4:** Specialist MDs contracted by the MOH, February 2004

**Contract & salary schemes**	**N**	**Average monthly salary (US$)**	**Origin**	**Fund source**	**Total annual cost (US$)**
Bilateral agreement	86	1000	Mainly Cuban	State budget	1 032 000
Topping up	19	1647**	Mozambican	SDC/CF	375 516
"Ex-Pooling"	32	2906	Mix	CF*	1 115 904
Standard individual contract	50	1300	Mix	State budget	780 000
Individual, the same as NHS	14	597	Mix	State budget	100 296
*Sub-Total*	201	1411	-	-	3 403 716
Nationals (NHS salary)	78	563	Mozambican	State budget	655 332
*Grand total*	298	1135	-	-	4 059 048

* Common fund

** Just topping up by donors. These doctors receive also a monthly average of about US$ 563 by the NHS, whose annual total cost can be found under 'NHS salary, total annual cost'.

*** Housing & travel allowances not included.

### Specialized MD costs and availability

Local Technical Assistance (TA) in Africa is becoming increasingly expensive [11]. At least in Mozambique, public salaries, after a long period of depreciation, are now improving and outweighing gains in productivity [12]. The total annual average cost in 2003 for an NHS specialized MD was already about 7000 US$ at a moment when the debate to 'decompress' salary scales in the public sector was gaining momentum [13,14]. Besides salary, other personnel-related benefits include housing and fuel subsidies, use of service cars, etc. These figures compare well with the salaries paid in more developed countries such as Cuba, Russia, Ukraine or India. As a matter of fact, emigration of Mozambican MDs to Europe until now has been limited, especially compared with that from the other Portuguese-speaking African countries [15].

In Mozambique, it is the limited availability of medical specialists which puts salaries relatively high. Moreover, their scarce number means a profitable market for the suppliers, because the demand for better, individual services is constantly increasing among the growing urban bourgeoisie.

As in many other countries [16], most Mozambican specialists moonlight, while still keeping a foot in the NHS as a way of establishing their reputation within private practice, while benefiting from public sector securities and advantages. As a matter of fact, until 2002 the topping up scheme often failed to fill all its posts, showing that better salaries were not sufficient to move enough national specialists from their positions in Maputo.

At present, the availability of newly trained national specialists is increasing, notwithstanding the shortage of young MDs and the scarcity of training hospital capacity for all but the most basic specialities. The State Medical School (UEM) has trained MDs at a discouragingly low pace. On average, from 1975 to 2003, 22 MDs were trained annually in Mozambique. Drop-out rates have been astonishingly high, as well as unit training costs. This was ascribed to many difficulties such as poor high school preparation, lack of books and bibliography, erratic follow-up by tutors in the teaching at Maputo central hospital and insufficient financial support. Students are also unsatisfied with the burden of lecturing and quality of teaching [17].

A curricular reform is now under way together with the hiring of new lecturers (mainly from Cuba), and in the next three years these figures are expected to rise to 50–60 MD/year. Figures will still grow to an average of 120 MD trained per annum after 2007, when a new, private University in the central town of Beira, a recent institution functioning since 2001, will begin to yield its first graduates.

Between 1991 and 2002, the average annual output of specialized Mozambican MDs inside and outside the country has been only 7. In 2003, 21 new MDs completed their post-graduation, and 70 more are expected to be trained between 2004 and 2007.

## Discussion

The salary gap between nationals and expatriates for vacancies outside the capital was already being closed by the topping-up scheme. Eventually, no substantial savings from the substitution of foreign MDs with national MDs should be expected. The international oversupply of MDs (a by-product of socialist regime health policy) floods the global market with a large supply of well-trained, disciplined and relatively cheap specialized cadres, who could be hired for reasonable salaries to work in countries where lack of MDs is still severe. It has been argued that "self-reliance is not the ultimate objective, in a world where all countries are competing for intellectual capital on a global basis, and when foreign aid is as much about knowledge as it is about money" [18].

Although medical students originate from all the country's provinces, once they are admitted by the State University, their expectation is to obtain a job in the capital city. This is the first of many 'pull factors' which will maintain the high concentration of MDs in Maputo, to where they will return once their two-year term of mandatory rural employment as junior doctors is completed [19]. In fact, as in many other developing countries, extra-earning possibilities, living and working conditions, career perspectives and professional improvement opportunities are far better in the capital city, for the beginner as well as for the most capable and experienced physicians. Other national MDs could migrate to the developed world (Portugal) or to neighbouring countries, such as South Africa and Botswana, where AIDS is taking a heavy toll on human resources for health [20]. Moreover, international NGOs managing AIDS programs will open new posts and opportunities for the health professionals in Mozambique. If economic growth in Mozambique keeps up the pace recorded in the first ten years following the civil war (implying a real GDP growth of 41.1% for the years 2004–2008 [21]), a considerable fraction of the new specialists will be absorbed by private practice, mainly in Maputo. Thus, the gap between the demand for, and the availability of, specialized MDs could increase in the next 5–10 years. From a public sector perspective, however, an increase in specialist MDs outside the capital must take into account their current work load and the possibilities of growth and re-qualification of the few existing referral health facilities. It must also be noted that specialist MDs are just marginally involved in supervising the peripheral network, initial training and on-the-job-training of medical assistants, nurses and midwives, despite the fact that these lesser skilled cadres are the backbone of the clinical activities, especially at the periphery of the health system.

The national political option to carry out a public sector decentralization policy could give rise to more individual contracts of national or expatriate MDs but, as it has been observed, decentralization has often remained a discourse, with civil service reform having been announced several times, but never really having materialized [22]. It should be central to an improvement in management capacity (a rather intricate issue to implement, as it proved in practice [23]) and to transferring substantial financial responsibility from the headquarters to the provincial health directorates and hospitals. At present, only the Maputo Central Hospital has enough money and competence to act autonomously. Private recruiting agencies acting worldwide could more efficiently provide value for money and the role of comprehensive bilateral agreements could be reduced. However, individual contracts managed directly by hospitals would entail higher transaction costs, particularly in the initial stages.

## Conclusion

Expatriate MD contracting in Mozambique has been a history of effective policy and also a rare example of sound pragmatism which prevailed against old-fashioned nationalist wishful thinking. The way a pooling mechanism supported by the donor community funded the salaries of high level expatriate medical cadres directly chosen by the recipient country, has been a positive, noteworthy experience. Allopathic medicine speaks the same language everywhere. Universally, medical universities offer remarkably homogeneous curricula; doctors update their knowledge through the same basket of international medical literature. In the global market the medical profession is rather easy to sell and purchase. When expatriate doctors learn the immigration country's language and health system rules, their integration is quickly achieved. For ordinary referral hospitals posts, lack of sophisticated diagnostic devices and expensive drugs make specialists from other developing countries more suitable to the local situation than their first-world counterparts, who are used to working in a more 'professional' environment, with technical support and at a cost too distant from what they could find in Mozambique. In global terms, a redistribution of the medical workforce, acting on economical incentives – from countries where it is excessive to countries where it is still scarce – makes sense and should be encouraged. In the end, at least in Mozambique, expatriate specialists do not cost significantly more than national ones. Increasing budget support by the donors should enable governments to recruit specialized MDs from countries where expertise is reasonably cheap, such as Cuba, the former Soviet Union, China and India. Thus, as a little contribution to alleviate the enormous health problems of Africa, a pattern of "poor"-to-"poor" cooperation, may be encouraged, supported financially by the wealthy North – a system that has proven to have been cost-effective in Mozambique.

The two different kinds of contracts for specialized MDs (through a bilateral government agreement and on individual basis), proved to be equally useful, offering balancing advantages to the health system. In the future, real willingness to decentralise responsibilities and competences, improvement in management capacity and cost considerations would be the factors favouring individual instead of collective contracting mechanisms for employment of expatriate MDs.

From a Mozambican perspective, a sustained increase of specialised MDs in the public sector outside the capital district would be fully meaningful only if it were in line with an upgrading of the health system as a whole (especially of the small intermediate portion made up of rural and district hospitals), and/or if these cadres were more thoroughly employed in activities of training and supervision. In the first case, the critical health policy issue would be about how and where to direct major investments financed from the scarce resources available. In the second one, an efficient way of coordinating activities of systemic supervision, on-the-job training of the supervised cadres and initial training must be found, which means for the most differentiate referral units to take responsibility for the medical care provided in all the health facilities within their catchment area. Otherwise, it would be nonsensical to hire expensive, well-qualified health cadres without adequately using their skills. A more balanced distribution, based on work load comparisons between hospitals of the same level, could also be useful in improving efficiency. Finally, the option of improving high-quality health services must be weighed up against the needs of expanding a primary health network which, at the moment, barely gives access to 50% of the population.

## Competing interests

The author worked as a technical advisor to the Human Resources Directorate, in the Ministry of Health of Mozambique from 2000 to 2004.

## Authors' contributions

Ferruccio Vio is entirely responsible for the final manuscript. The views expressed in the paper are those of the author only and do not necessarily represent the view of the Ministry of Health.
